# Ionic Liquid-Based Thermoplastic Solid Electrolytes Processed by Solvent-Free Procedures

**DOI:** 10.3390/polym10020124

**Published:** 2018-01-26

**Authors:** Francisco González, Víctor Gregorio, Aitor Rubio, Leoncio Garrido, Nuria García, Pilar Tiemblo

**Affiliations:** Instituto de Ciencia y Tecnología de Polímeros, ICTP-CSIC, Juan de la Cierva 3, 28006 Madrid, Spain; fgonzalez@ictp.csic.es (F.G.); victor.gregorio.gregor@gmail.com (V.G.); aitorrubioro@hotmail.com (A.R.); lgarrido@cetef.csic.es (L.G.)

**Keywords:** polymer electrolytes, thermoplastic electrolytes, solid electrolytes, ionic liquids, energy storage

## Abstract

A series of thermoplastic polymer electrolytes have been prepared employing poly(ethylene oxide) (PEO) as a polymer matrix, bis(trifluoromethane sulfonimide) (LiTFSI), and different room-temperature ionic liquids (RTIL) with bis(fluorosulfonyl)imide (FSI) or TFSI anions. This formulation makes them safe and non-flammable. The electrolytes have been processed in the absence of solvents by melt compounding at 120 °C, using sepiolite modified with d-α-tocoferol-polyethyleneglycol 1000 succinate (TPGS-S) as a physical cross-linker of PEO. Several concentrations of RTILs, lithium salt, and TPGS-S have been tested in order to obtain the highest ionic conductivity (σ) without losing electrolytes’ mechanical stability. The materials’ rheology and ionic conductivity have been extensively characterized. The excellent crosslinking ability of TPGS-S makes the electrolytes behave as thermoplastic materials, even those with the highest liquid concentration. The electrolytes with the highest concentrations of FSI anion present a σ over 10^−3^ S·cm^−1^ at 25 °C and close to 10^−2^ S·cm^−1^ at 70 °C, and notably behave as solids at temperatures up to 90 °C despite over 65 wt % of their formulation being liquid. The electrolytes thus obtained are safe solid thermoplastics prepared by industrially scalable procedures and are suitable for energy storage devices, proving the adequacy of polymer-based materials as solid electrolytes for batteries or supercapacitors.

## 1. Introduction

Energy storage is one of the main items on the current scientific and technological agenda. Polymers have gained prominence as both as electrode and electrolyte materials. In particular, the possibility they offer to produce solid electrolytes with adequate mechanical and transport properties is unique. Employing Solid Polymer Electrolytes (SPE) in electrochemical batteries offers many advantages in comparison to liquid ones, and a main drawback, which is the low ionic conductivity inherent to their solid state. Numerous studies can be found in the scientific literature [1,2] on the different ways to prepare SPEs with the highest possible ionic conductivity (σ) and sufficient mechanical stability. These strategies are mostly based on making the electrolyte behave as a liquid at the microscopic scale, so as to increase ionic mobility. For example, employing working temperatures where the electrolyte becomes a viscous liquid [3] or introducing large ratios of a liquid component [4,5]. Some of the polymer electrolytes proposed involve the generation of chemically crosslinked 3D networks to make the electrolyte macroscopically solid while microscopically liquid, a strategy that does not have the many advantages of a thermoplastic polymer. A solid thermoplastic SPE, i.e., an electrolyte that can be extruded or injected, and that can be reshaped, reprocessed and recycled, is indeed a very challenging endeavor. Such a material would allow not only the convenient extrusion of electrolytes but would also serve as a material for printing flexible batteries in 3D printers. However, no thermoplastic solid electrolyte offers the optimum balance between safety, ionic conductivity, and mechanical stability.

Recently, SPEs composed of thermoplastic polyurethane (TPU), poly(ethylene oxide) (PEO) and lithium bis(trifluoromethane sulfonimide) (LiTFSI), with σ = 5.3 × 10^−4^ S·cm^−1^ at 60 °C have been reported [6]. They were prepared by dissolving TPU and PEO/LiTFSI in different solutions of *N,N*-Dimethylformamide (DMF). However, solvent-free procedures are more convenient because of environmental and health issues. As examples of solvent-free or green SPEs, blends of comb-like nonionic water-borne polyurethane (NWPU) with LiClO_4_ in aqueous dispersion [7] and poly(ethylene oxide)-copoly(propylene oxide) copolymer cross-linked by a bisphenol-A diglycidyl ether, LiClO_4_ and (1-ethyl-3-methylimidazolium bis(trifluoromethanesulfonyl)imide) EMITFSI as liquid phase were synthesized. These last electrolytes present a σ = 1.38 × 10^−3^ S·cm^−1^ at 20 °C and were used for electric double-layer capacitors [8] These interesting electrolytes are, however, not thermoplastic, being chemically crosslinked.

Room-Temperature Ionic Liquids (RTILs) like EMITFSI are extremely interesting as components in electrolytes. Besides their low flammability, which contributes to their safe use, some RTILs have proved to have other advantageous features as electrolytes, namely the ability to form a solid electrolyte interphase (SEI) able to suppress the formation of Li dendrites in the case of Li batteries [9,10,11]. For this purpose, the combination of such RTILs with a polymer to form a solid electrolyte seems very appealing. Unfortunately, RTILs’ high viscosity lowers their conductivity as compared to similar electrolytes containing ethylene (EC) or propylene (PC) carbonates. Some time ago we reported on highly conductive physically crosslinked electrolytes [12,13], processed by melt compounding using PEO, LiTf, EC, and a small amount of an ad hoc modified sepiolite (named TPGS-S) acting as a physical cross-linker [14]. These electrolytes are solid up to 90 °C and show ionic diffusivities similar to those of a chemically analogous viscous liquid, i.e., the materials’ design allowed the decoupling of the rheological macroscopic properties and the microscopic ionic mobility. The electrolytes, prepared by solvent-free procedures, are thermoplastic, and possess remarkable electrochemical stability [15]. Aware of the fact that carbonates should be avoided in favor of safer liquids, we attempted the preparation of analogous electrolytes but substituted the EC liquid fraction by a set of RTILs. This was successfully done [16], in the sense that RTIL-containing, safe thermoplastic electrolytes were prepared with dimensional stability up to 90 °C, but with the RTIL being of higher viscosity than carbonates, the σ values were clearly lower (≈5 × 10^−4^ S·cm^−1^ at 25 °C at best). σ also suffered from the fact that LiTf was employed, while LiTFSI now appears to be a much better choice. The current background on the performance of RTILs as electrolytes with and without PEO [1,17,18,19,20] shows that in many electrochemical applications, the incorporation of batteries, capacitors, supercapacitors, or pseudocapacitors in the formulation of electrolytes is a worthy challenge, and hence we undertook the objective of producing mechanically tough thermoplastic electrolytes based on RTILs and PEO with the highest possible liquid fraction, and hence the highest possible conductivity, both with and without Li salt. This work collects the efforts done in this direction, and the results obtained.

## 2. Materials and Methods

### 2.1. Materials

PEO *M*_w_ = 5 × 10^6^ g·mol^−1^ from Sigma-Aldrich (Sigma-Aldrich, St. Louis, MO, USA) was used to prepare the composites. LiTFSI, from Aldrich and neat sepiolite, kindly supplied by TOLSA S.A. (TOLSA, Madrid, Spain), were dried under a vacuum for 24 h. d-α-tocopherol polyethylene glycol 1000 succinate (TPGS), used to prepare the modified sepiolite TPGS–S, was purchased from Aldrich and used as received. Details on the preparation of TPGS-S have appeared elsewhere [14]. The RTILs employed to prepare the electrolytes listed in Table 1 were purchased from Solvionic (Solvionic, Toulouse, France), all of them with 99.5% purity. They are the following: 1-ethyl-3-methylimidazolium bis(fluorosulfonyl)imide (EMIFSI), *N*-propyl-*N*-methylpyrrolidinium bis(fluorosulfonyl)imide (PMPFSI); *N*-propyl-*N*-methylpyrrolidinium bis(trifluoromethanesulfonyl)imide (PMPTFSI), 1-ethyl-3-methylimidazolium bis(trifluoromethanesulfonyl) imide (EMITFSI).

Solutions of LiTFSI with RTILs and poly(ethylene glycol) methyl ether (PEG), *M*_n_ = 550 g·mol^−1^ from Sigma-Aldrich (Sigma-Aldrich, St. Louis, MO, USA) were prepared by magnetic stirring for 30–120 min, as model liquid phases for the solid polymer electrolytes. 

### 2.2. Preparation of the Composite Electrolytes

The electrolytes’ composition appears in Table 1. The components were melt-compounded in a Haake MiniLab extruder (Thermo Fisher Scientific, Waltham, MA, USA) at 80 rpm, at different residence times and temperatures, as listed in Table 1. The conditions employed ensure that minimal degradation of PEO will occur. Processing conditions were varied to study their effect on the electrolytes’ features and properties, by decreasing processing time and temperature and incorporating an additional step consisting of premixing of the RTIL, LiTFSI, and TPGS-S by magnetic stirring for 10 min.

Electrolytes have been denoted according to the RTIL used and ranked from lowest to highest RTIL concentration; some of the electrolytes have been prepared with 5 wt % of TPGS-S and others with 2.5 wt %, in an attempt to reduce to a maximum the solid phases in the electrolytes (in favor of liquid ones) while preserving the solid-like behavior of these materials. 

### 2.3. Characterization

Characterization of electrolytes was done on films of controlled thickness (~500 and 1000 μm for rheological measurements), processed by compression molding at 75 °C during 3 min. 

Scanning electron microscopy (SEM) was performed with a Hitachi SU-8000 (Hitachi, Ltd., Tokyo, Japan). Samples were fractured after immersion in liquid nitrogen and the sections were observed unmetalized. 

Differential scanning calorimetry (DSC) studies were performed in a TA Instruments Q100 (TA Instruments, New Castle, DE, USA). The heat flow was recorded as follows: a first cooling–heating cycle at 10 °C·min^−1^ from 120 °C to −60 °C, followed by a second cooling–heating cycle from 120 °C to −80 °C at −20 °C·min^−1^. The DSC curves represented in this work are those recorded in the second cycle. 

ATR-FTIR. IR spectra were recorded on the surface of the electrolytes using a FTIR Perkin-Elmer Spectrum-One (PerkinElmer, Waltham, MA, USA), with 10 scans and resolution of 4 cm^−1^. 

Determination of diffusion coefficients (D) was done by ^7^Li and ^19^F PFG-NMR in a Bruker Avance^TM^ 400 spectrometer (Bruker BioSpin GmbH, Rheinstetten, Germany) equipped with a 89 mm wide bore, 9.4 T superconducting magnet (Larmor frequencies of ^7^Li and ^19^F at 155.51 and 376.51 MHz, respectively). The ^7^Li and ^19^F diffusion data were acquired at 25 ± 0.1 °C with a Bruker diffusion probe head, Diff60, using 90 ° radiofrequency (rf) pulse lengths of 11.0 μs. The diffusion experiments were done as described before [16].

Rheological measurements were performed using an Advance rheometer AR G2 (TA Instruments, New Castle, DE, USA) with a 20 mm steel plate. The 1000 μm thick films were prepared by compression molding at 90 °C for 3 min and cooled down to room temperature quickly. Prior to the launching of the experiment, samples were annealed in the rheometer for 10 min at the extrusion temperature (120 °C) and then stabilized at 75 °C for 5 min. Oscillatory frequency sweeps were performed in the frequency range of 500–0.01 rad·s^−1^ using a 10 Pa stress amplitude, which lies within the linear viscoelastic regime for the samples tested. 

Creep experiments were done as follows: electrolytes’ films were sandwiched between two gold electrodes of 20 mm of diameter, and placed on a heating plate with 0.5 kg on top at 70 and 90 °C for 20 min at each temperature. Some electrolytes were tested for longer periods of 10 months enduring 0.1 kg weight at room temperature. All electrolytes containing TPGS-S endure these experiments without creep being noticed, and all can be considered solid-like. 

The viscosity of RTILs and their mixtures with LiTFSI and PEG were measured using an Anton Paar Stabinger Viscometer SVM^TM^ 3000 (Anton Paar GmbH, Graz, Austria) in a temperature range from 10 to 70 °C.

Conductivity of the electrolytes was determined in a NOVOCONTROL Concept 40 broadband dielectric spectrometer (Novocontrol Technologies GmbH, Montabaur, Germany) in the temperature range −50 °C to 90 °C in the frequency range 0.1 and 10^7^ Hz. Disk films of dimensions of 2 cm diameter and ~500 μm thickness were inserted between two gold-plated flat electrodes, then a frequency sweep was done every 10 °C, cooling to −50 °C and then heating to 90 °C; thereafter, the same measurements were done but cooling from +85 to 25 °C. σ of the samples was calculated by using conventional methods based on the Nyquist diagram and the phase angle as a function of the frequency plot, as described before [12]. The values of σ that appear in this work correspond to the second heating σ measurement. 

**Scheme 1:**
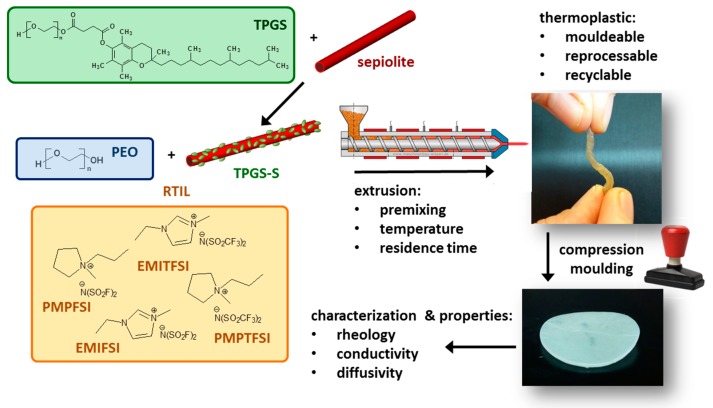
Methodology of the preparation of the thermoplastic electrolytes by melt-compounding.

Scheme 1 summarizes the methodology followed to prepare the electrolytes described in this work.

## 3. Results and Discussion

In the search for highly conductive thermoplastic electrolytes, high concentrations of RTILs or LiTFSI/RTIL have been employed in combination with PEO, as shown in Table 1. In Table 2 we give the composition, crystallinity of the electrolyte in wt % (χ_c_), glass transition temperature (*T*_g_), and σ at 25 °C. The electrolytes are divided into four groups according to the RTIL employed. Components with good mixing ability have been chosen. TPGS-S is added, as in a previous work [16], to confer solid-like characteristics to the electrolytes. Details on the different preparation procedures and nomenclature appear in the experimental section. 

In many of the electrolytes of Table 2, the liquid phase RTIL + Li salt amounts for about 66 wt % of the electrolyte. Therefore, it was considered worthwhile to characterize the viscosity (η) and melting temperatures (*T*_m_) of model liquid phases representing the electrolytes’ liquid phase; they appear in Table 3. The effect of PEO is modeled by adding PEG instead.

While all the RTILs employed are crystalline and melt in the *T* range −16 °C (EMIFSI) to 12 °C (PMPTFSI), of the binary mixtures with salt only, PMPTFSI + LiTFSI is still able to crystallize under the experimental conditions employed in this work. It shows several melting peaks, from about 7 °C to −18 °C, and freezing temperatures of about −40 °C. Note that PMPTFSI is the RTIL with the highest melting point (12 °C), and that the addition of LiTFSI produces a strong freezing-point depression; then, very probably, the freezing temperatures of EMIFSI + LiTFSI, EMITFSI + LiTFSI, and PMPFSI + LiTFSI are too low for these mixtures to crystallize in the experimental conditions employed in this work, where the minimum experimental temperature in DSC measurements is −80 °C. Finally, none of the ternary mixtures with PEG crystallizes.

**Figure 1:**
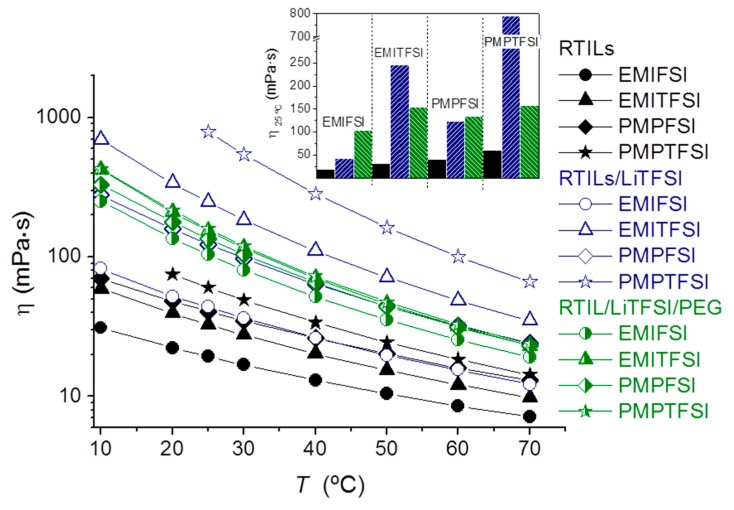
η dependence on temperature for RTILs (black) and solutions with LiTFSI (blue) and LiTFSI + PEG (green). The inset shows the viscosity at 25 °C divided into groups attending to the RTIL nature (color code as before).

The η of the different model liquid phases as a function of temperature have been measured and appear in Figure 1. For binary mixtures of RTILs/LiTFSI, η increases dramatically with respect to pure RTILs, especially for RTILs with TFSI anions, following the η order EMIFSI << PMPFSI << EMITFSI << PMPTFSI. This is expected as FSI is known to lead to much lower η increases upon adding Li^+^ than TFSI [21]. The mixture PMPTFSI/LiTFSI, which has a melting peak at about 7 °C, becomes too viscous to be measured (under the conditions used in this work) below 25 °C.

When PEG is added to the RTIL/LiTFSI mixture, a η decrease is seen in RTILs with TFSI anion, i.e., those where the addition of LiTFSI had caused a very large η increase. This η decrease can be explained by the ability of PEG molecules to complex Li^+^, and “remove” it from the RTIL solution. Besides, in the PMPTFSI/LiTFSI/PEG mixture no crystalline phases are seen, which will make its viscosity lower than that of the PMPTFSI/LiTFSI mixture. In turn, the increase of η produced by the addition of PEG in the solutions of LiTFSI in RTILs with FSI is simply due to the higher η of pure PEG with respect to these RTIL/LiTFSI mixtures. This results in very similar η and η(T) for all the model phases regardless of the RTIL nature. 

The study of the model liquid phases offers very interesting information. First, there is a decrease in *T*_c_ on adding LiTFSI to any of the RTILs. Second, as diffusivity is inversely proportional to η (if the Stokes–Einstein equation holds), then if the electrolytes with the different RTILs behave as the model phases with PEG, the ionic diffusivity will be similar in all of them, because η in the ternary mixtures varies little, only from 104 to 158 mPa·s at 25 °C. However, if the electrolytes with different RTILs behave like the model phases without PEG, the ionic diffusivity will be very different in all of them because η in the binary mixtures varies strongly, from 44 to 790 mPa·s at 25 °C. These two Δη limits mark the two limit morphologies of the electrolytes, from a complete mixing (which would resemble the ternary mixture behavior) to a complete phase separation (featured by the binary mixture). 

### 3.1. Physicochemical Characterization of the Electrolytes

**Figure 2:**
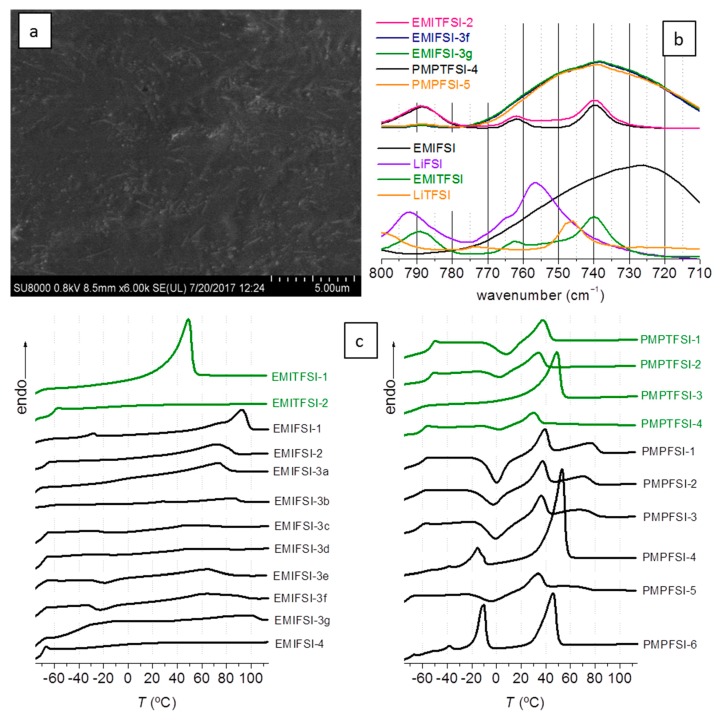
(**a**) EMIFSI-3c SEM image; (**b**) FTIR of some electrolytes (up) and RTIL and salts (down); and (**c**) DSC scans of a set of electrolytes.

Figure 2a is a SEM image of EMIFSI-3c where abundant isolated TPGS-S can be seen. This is how TPGS-S appears in the electrolytes processed for 20 min and premixed. SEM imaging also indicates (not shown) that few or no crystalline morphologies are detected in these electrolytes, contrary to what happened in electrolytes with less [RTIL] [16], where morphologies related to crystalline order were seen in all electrolytes except those prepared with EMIFSI. In Figure 2b the FTIR spectra of the υ(S-N) region of FSI and TFSI in several electrolytes appear. This vibration is related to the aggregation state of the anions [22]. In the lower half, the same region is shown for the pure compounds EMIFSI, LiTFSI, EMITFSI, and LiFSI. The electrolytes that contain only the TFSI anion show a spectrum almost identical to that of TFSI as in EMITFSI (mild interaction of TFSI with the cation, either EMI or PMP), and the bands corresponding to TFSI, as in LiTFSI (strong interaction between TFSI and the cation), are not seen. This is because, when LiTFSI is added to the mixture of ionic liquid and PEO, Li becomes preferentially coordinated by the PEO chain, leaving TFSI free to become involved in milder interactions with the cations of the ionic liquid. In the electrolytes with a mixture of FSI and TFSI anions, the spectral region is broad and a mixture of species corresponding to TFSI as in EMITFSI (739 cm^−1^) and FSI as in EMIFSI (or PMPFSI) can be suspected. Again, Li is dissolved by the PEO chain, and the anions TFSI and FSI interact with the ionic liquid cation (EMI or PMP). These spectra suggest that the liquid phase in the electrolytes is closer to the model liquid phases prepared with PEG than to those without PEG (see Figure 1), and so no ionic liquid crystallization is to be expected. Moreover, no extreme differences in viscosity are to be expected either.

In Figure 2c the DSC scans of the electrolytes are collected; in general they show complex multiphasic materials. Electrolytes with EMIFSI liquid tend to be less crystalline than analogue electrolytes with the other RTILs. The PEO melting endotherm, which, when pure, appears close to 60 °C, and involves over 60 wt % of the polymer, is well seen in PMPFSI-4, PMPFSI-6, EMITFSI-1, and all PMPTFSI between 40 and 60 °C, but it is imperceptible or almost so in EMIFSI-3c, EMIFSI-3f, EMIFSI-4, and EMITFSI-2. Compare, for example, the χ_c_ of EMIFSI-2 with PMPFSI-1, PMPFSI-2, or PMPFSI-3. Table 2 also shows that the higher the [RTIL], the lower the χ_c_ (irrespective of the RTIL).

In some of the electrolytes, especially those prepared with EMIFSI, it is possible to see a small melting endotherm at *T* > 60 °C (PEO melting temperature), ranging from 75 to 100 °C. This endotherm is more conspicuous in the first scanning than in subsequent ones, as if this crystalline phase requires time to develop. It is very clear in EMIFSI-1 (which is the most crystalline electrolyte among those prepared with EMIFSI), but exists in most of the electrolytes prepared with EMIFSI, at least on the first scan. It is also seen in three electrolytes prepared with PMPFSI, PMPFSI-1, PMPFSI-3, and PMPFSI-5, and does not appear in the electrolytes prepared with PMPTFSI or EMITFSI. This suggests that this high T melting peak is related to a phase containing PEO and FSI. 

As for the low-temperature region, Figure 2c shows that many of the electrolytes seem to be one or several *T*_g_ under that of pure PEO, even at *T* < −60 °C. This is caused by the interaction of the salt LiTFSI and the RTIL with PEO, and reveals that there is compatibility among the components. It is known that Li salts and LiTFSI in particular plasticize the PEO chain [23]. In those with the largest concentration of RTIL + LiTFSI, however, no *T*_g_ is seen. The exception is EMIFSI-3g, which has a very short residence time in the extruder, where the PEO *T*_g_ is seen at about −41 °C, suggesting the existence of a PEO-rich phase.

Given the complexity of the composition (at least three and usually four components in each electrolyte) and the multiphasic character of some of the components (PEO has a *T*_m_ ≈ 60 °C and *T*_g_ ≈ −40 °C, the RTILs have *T*_m_ between −16 °C and 12 °C) it is not surprising that their blends display numerous phases. As explained before in relation to the model liquid phases, the final heterogeneity of the electrolytes will depend on how compatible the components are and how complete the mixing has been. Considering the basic features of polymer blending, the well-known unlikeliness of thermodynamic mixing, and their high viscosity, the quality of mixing and hence the final morphology of these electrolytes depends very strongly on the processing conditions. This is relevant in these materials due to their ultimate purpose of becoming industrially scalable electrolytes.

To summarize, these electrolytes are little crystalline, especially the EMIFSI-containing ones, and display a complex phase distribution in the T range −80 °C to 100 °C that is strongly dependent on the mixing quality, i.e., on processing conditions. SEM imaging shows good dispersion of the TPGS-S fibers and FTIR reveals that in all of the electrolytes, complexation of Li by the PEO chain occurs extensively.

### 3.2. Rheology

**Figure 3:**
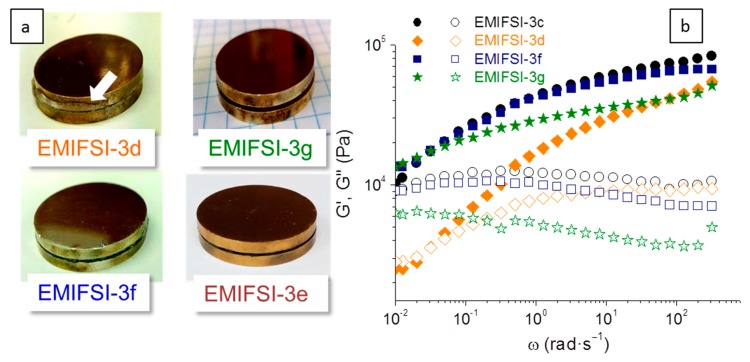
Rheology of the polymer electrolytes: (**a**) Pictures of the sandwiched electrolytes EMIFSI-3d, f, and g after enduring the creep text consisting of 0.5 kg for 20 min at 75 °C and then 20 min at 90 °C (see text for details). Picture of sandwiched EMIFSI-3e electrolyte after 10 months with 0.1 kg on top, and (**b**) shear storage G′ (solid) and loss G″ (open) moduli at 75 °C for EMIFSI-3c, d, f, and g.

In an attempt to reduce the solid phases in the electrolytes (in favor of liquid ones), the amount of TPGS-S has been reduced from 5 wt % [16] to 2.5% in some of the electrolytes. Additionally, the effect of sepiolite organic modification has been checked by blending a sample with pure sepiolite (EMIFSI-3d) instead of TPGS-S. Then, a rheological study was done, which is summarized in Figure 3, where the effect of [RTIL] and processing conditions is studied. The effect of TPGS-S instead of pure sepiolite S is illustrated with a simple creep experiment, described in detail in the experimental section, which basically consists of placing the electrolytes between the electrodes to endure 0.5 kg for 20 min at 75 °C and then at 90 °C. After this experiment, EMIFSI-3d, which contains pure sepiolite, showed signs of creep, while the other samples did not. Pictures showing the appearance of the sandwiched electrolytes, EMIFSI-3d, EMIFSI-3f, and EMIFSI-3g, after this creep test are shown in Figure 3a. This is noteworthy as EMIFSI-3f and EMIFSI-3g contain only 2.5 wt % of TPGS-S, and EMIFSI-3g has been processed for only 4 min, instead of 20 min as with the rest of the samples. This different processing means the mixing is not so homogeneous (as shown in the DSC) but, interestingly, EMIFSI-3g is still solid-like. From a practical viewpoint, and having industrial scalability in mind, the fact that solid-like electrolytes can be made by melt-compounding in such a simple and quick way is highly interesting. 

In columns 4 and 5 of Table 2, the values of G′ at 75 °C and 0.05 rad·s^−1^ and the frequency at which G′ = G″ are collected. A clear effect of premixing on the solid character of the samples is seen. We see that all non-premixed electrolytes (irrespective of nature and concentration of the RTIL) show lower G′ values and crossover (G′ = G″) frequencies ω > 0.01 rad·s^−1^, while all premixed samples display no crossover up to ω = 0.01 rad·s^−1^ and have G′ values over 2-fold higher (even EMIFSI-3g with very low extrusion time). This is certainly caused by the different quality of the TPGS-S distribution in the electrolytes, differences that are not detectable in SEM imaging. Premixing is, as regards rheology, the key to solid-like behavior.

In the pre-mixed EMIFSI-3n series, an additional creep experiment was done consisting of withstanding 0.1 kg at RT for 10 months. In none of the electrolytes tested was creep noticeable. Figure 3a includes the picture of EMIFSI-3e after this test. The rheological measurements provide quantitative evidence of this pseudosolid behavior. Figure 3b shows the shear storage (G′) and loss (G″) moduli variation with frequency at 75 °C for the set of pre-mixed electrolytes with the highest [RTIL]. EMIFSI-3d with pure sepiolite instead of TPGS-S has a lower G″ and G′ moduli than the other three samples. For ω < 0.02 rad·s^−1^ G″ > G′, i.e., for the lower frequencies, EMIFSI-3d is behaving like a liquid. This does not occur for the electrolytes containing TPGS-S, EMIFSI-3c, EMIFSI-3f, and EMIFSI-3g, which present a higher G′ than G″ in the whole frequency range; most importantly, no crossover is seen, confirming their solid behavior at 75 °C. 

### 3.3. Ion Conductivity and Diffusivity

**Figure 4:**
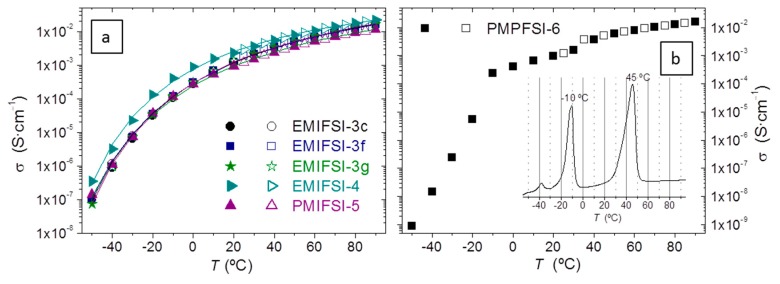
(**a**) σ dependence on temperature during the heating and cooling cycles. The lines are the VFT fitting in Table 4. (**b**) Comparison between σ and DSC curve for PMPFSI-6 electrolyte. The inset shows the DSC trace for this electrolyte.

Table 2 contains σ of the different electrolytes at 25 and 70 °C, and Figure 4 represents the variation of σ with temperature in the range −50 °C to 90 °C of a selection of electrolytes. It is remarkable that, though the DSC in Figure 2 shows the phase heterogeneity of the electrolytes, this has, as a rule, little effect on σ, as seen for instance in EMIFSI-3c, EMIFSI-3f, and EMIFSI-3g in Figure 4a—all very similar as regards σ(T) in spite of the different low T phase distribution evidenced in their DSC. Another indication that phase transitions or relaxations are not being reflected in σ(T) is that the σ measurements performed on cooling from 90 °C or on heating from −80 °C coincide absolutely. Moreover, all electrolytes in Figure 4a can be fitted with a single Vogel–Fulcher–Tamman (VFT) equation, $\ln\left(\sigma \right)=\ln\left(\sigma_{\infty }\right)-\frac{B}{{T-T}_{0}}$ (Equation (1)), where σ_∞_ (S·cm^−1^), B (K), and T_0_ are constants. The fittings are very good, indicating that the σ(T) dependence is characteristic of a viscous liquid in which σ is governed by η in the whole temperature range, and the VFT fitting of σ(T) reflects the VFT variation of η(T). The fitting parameters appear in Table 4. Minor variations are obtained for T_0_ and B parameters, related to the activation energy in the samples EMIFSI-3n, indicating that the different processing conditions employed in these electrolytes do not affect σ(T). The main differences between electrolytes with EMIFSI/LiTFSI and PMPFSI/LiTFSI are σ_∞_, with the latter being less conductive at high temperatures, which is consistent with the lower η of EMIFSI and its mixtures in Table 3. The fitting of EMIFSI-4 σ results in a set of different parameters because this electrolyte has no Li salt.

Almost all the electrolytes in Table 2 behave like those in Figure 4a, in the sense that no phase transitions are seen to affect σ(T). The exceptions are EMITFSI-1and PMPFSI-6, both without Li salt and a large fraction of RTIL. PMPFSI-6 appears in Figure 4b: σ measured on heating and on cooling do not coincide, being slightly higher when measuring on cooling from the melt. In PMPFSI-6 σ does not follow a VFT decrease with T, but decreases very quickly at about 40 °C and −10 °C. The PMPFSI-6 DSC scan in the inset of Figure 4b has two strong melting endotherms, one at about 40 °C and another at about −10 °C. PMPFSI-6 is crystalline in about a 20%. The transition at 40 °C is the melting of the PEO crystallites, while that at 10 °C is the PMPFSI melting (Table 3). Thus, phase transitions involving large fractions of the electrolyte do appear as variations in σ(T). 

Besides EMITFSI-1 and PMPFSI-6, there is a third electrolyte prepared without Li salt and a large [RTIL], which is EMIFSI-4. Contrary to EMITFSI-1 and PMPFSI-6, the σ(T) of EMIFSI-4 can be fitted with a VFT equation in the whole T range (see VFT parameters in Table 4, suggesting that no phase transitions are occurring. It seems then that when the RTIL is EMIFSI, PEO finds it more difficult to crystallize, probably because of a larger interaction between the polymer chain and the RTIL.

**Figure 5:**
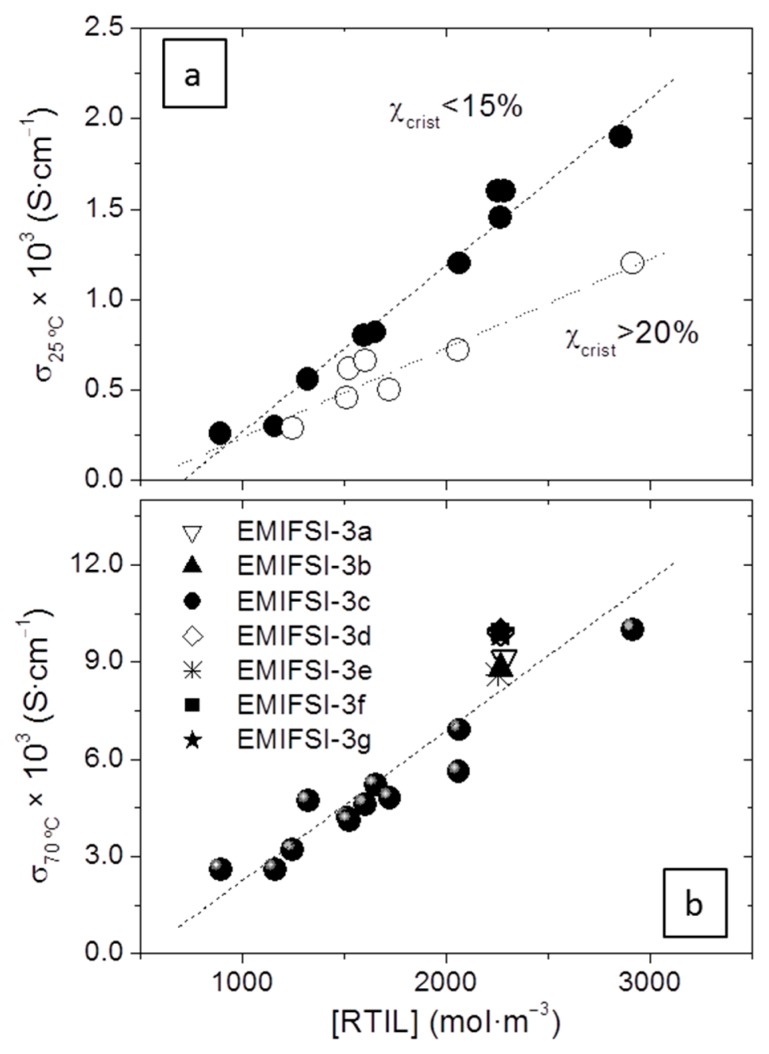
σ dependence on [RTIL] for solid electrolytes at 25 °C (**a**) and 70 °C (**b**).

Figure 5 shows the representation of σ as a function of [RTIL] under the PEO melting point, at 25 °C (in Figure 5a), and above it, at 70 °C (Figure 5b). Even if phase transitions are too mild to be detected in the σ(T) curves of Figure 4, at 25 °C the presence of a certain amount of crystallinity divides the electrolytes into two groups, ones with χ_c_ < 15% (more conductive for the same [RTIL]), and ones with χ_c_ > 20%, which are less conductive. The first group comprises the electrolytes prepared with EMIFSI, EMITFSI-2, and most of those prepared with PMPTFSI, while the second group comprises most of the electrolytes prepared with PMPFSI, EMITFSI-1, and PMPTFSI-3. The most conductive electrolytes at 25 °C reach values close to 2 × 10^−3^ S·cm^−1^, remarkable for a solid electrolyte, and about 4-fold higher than those reported previously in similar electrolytes with lower [RTIL] [16]. Of course, at 70 °C, when most of the crystallites have melted, the difference between the two groups disappears and though scatter is important (and expectable because of the different formulations) there is a very clear trend with the [RTIL], which is the most important factor in their overall σ. At 70 °C the solid electrolytes reach a remarkable σ ≈ 0.01 S·cm^−1^.

The fact that the RTIL concentration is more determinant than the RTIL nature as regards the σ values seems to suggest that the electrolytes are behaving like the liquid model phases including PEG, rather than those model liquid phases not including PEG, as if the latter was the case very large variations of σ (caused by very large variations of η, see Figure 1) would be observed and all σ would be clearly lower. That the electrolytes behave more like the PEG-containing model liquid phases is not surprising, and also suggests that Li is trapped in the PEO chain rather than moving solvated by FSI or TFSI anions, in accordance with the FTIR results in Figure 2b.

Figure 5b shows how different processing conditions (EMIFSI-3b to EMIFSI-3g) produce a certain scatter in σ. Electrolytes EMIFSI-3a, 3b, and 3e, where no premixing was done, have slightly lower σ than EMIFSI-3c, 3d, 3f, and 3g, in which premixing was done. Those values obtained in electrolytes that were not premixed are closer to the line that fits all the electrolytes, where no premixing was done, which is very reasonable. Those that were premixed show more reproducible and slightly higher values of σ. In any case, values differ by less than 15%. This small variation is probably caused by the larger tortuosity of the electrolytes in which mixing has been less efficient. For polymer-based electrolytes such as those studied in this work, which we hope will become a real solution at an industrial scale, this scarce effect of different processing conditions in remarkable σ at and over room temperature is a most important issue, for multiphasic systems highly sensitive to processing conditions are very difficult to scale up. 

Insight into the ionic transport characteristics of these electrolytes at a microscopic level is provided by the diffusion coefficients (D), which appear in Table 2. The diffusivity of the anions is outstanding, bearing in mind that they are solid materials up to *T* > 75 °C. In effect, D_TFSI_ or D_FSI_ of about 10^−11^ m^2^·s^−1^ at 25 °C are closer to a liquid’s than to a solid’s transport behavior. This is not surprising for many of the electrolytes have *T*_g_ < −40 °C and almost no crystallinity (Table 2), and as a consequence they are liquids at a microscopic level. The fact that they behave as solids up to *T* > 75 °C accounts for the excellent performance of TPGS-S as a physical cross-linker. 

Making use of the data in Table 2 and the Nernst–Einstein equation (Equation (2)), $\sigma_{NE}=\frac{F^{2}}{RT}\cdot \sum_{i}n_{i}\alpha_{i}\cdot D_{i}$, where n_i_ and D_i_ are the molar concentrations of the ion and its diffusion coefficient, respectively, it is possible to calculate the maximum conductivity if all the dissociation coefficients α_i_ were 1, which we have called σ_NE_ (column 8 of Table 2). It has not been possible to measure the cation in the RTIL by NMR and so it has been estimated, according to the literature [24], to be about 10% higher than FSI in the case of EMI and 10% lower in the case of PMP. The contribution of Li^+^ to σ_NE_, σ_NE_(Li) has also been calculated and included in column 7 of Table 2. 

**Figure 6:**
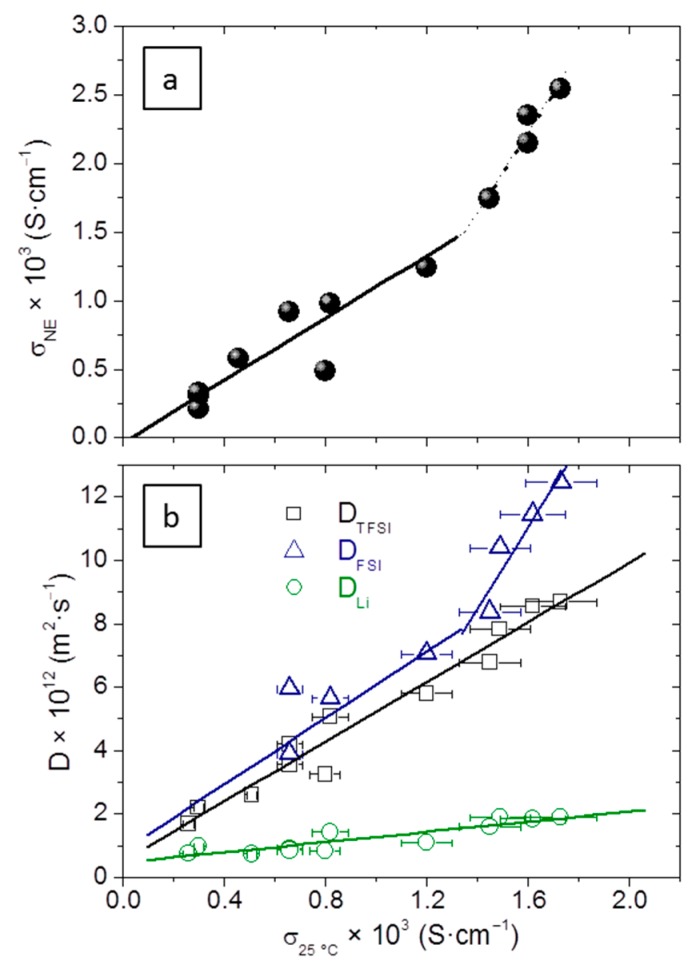
(**a**) Relationship between experimental σ at 25 °C and that obtained by the Nernst–Einstein equation (σ_NE_). (**b**) Relationship between D_Li_, D_TFSI_, D_FSI_, and experimental σ at 25 °C.

For a simple visualization of the differences between experimental and calculated σ, see Figure 6a. Though scatter is important (R = 0.98), a linear relationship is still seen up to σ ≈ 1.2 × 10^−3^ S·cm^−1^, the slope of which is *m* = 1.15, meaning that the average dissociation coefficient or ionicity of the electrolytes, α, which is the inverse of the slope, is about 0.87. This includes the LiTFSI salt dissociation and that of the RTILs employed. Pure RTILs such as those employed in this work have α in the range 0.5 to 0.7 at 25 °C, and LiTFSI is highly dissociated in the presence of PEO; this calculated value of α, though very high, is reasonable. Over σ ≈ 1.2 × 10^−3^ S·cm^−1^ a second linear relationship exists, with a higher slope, i.e., apparently lower overall ionicity. The electrolytes involved in this second trend all have the same formulation (with the highest [RTIL] used in this work) and differ only in the processing conditions, for they are the EMIFSI-3n set. 

Figure 6b shows the variation of the three diffusion coefficients D_Li_, D_TFSI_, and D_FSI_, as a function of σ. This illustrates how the contribution of Li to σ is much lower than that of the anions; as σ increases, it becomes progressively lower since the slope of D_Li_ is smaller than that of the anions. The increase of RTIL concentration makes the anions diffuse more quickly, to a much higher extent than Li^+^. This agrees with the preferential motion of Li^+^ in the PEO chain.

A single linear correlation is seen between σ and D_Li_ and D_TFSI_ for all the electrolytes measured, but a strong positive deviation is seen in the D_FSI_ of the EMIFSI-3n set. This explains the existence of a second linear relationship with a higher slope between σ_NE_ and σ in Figure 6a, because the large D_FSI_ that makes σ_NE_ increase is not contributing to the experimental σ. However, the reason why D_FSI_ is increasing in such a way in the EMIFSI-3n is not straightforward.

**Figure 7:**
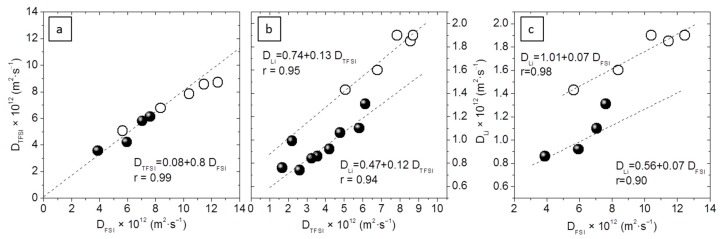
Relationship between (**a**) D_TFSI_ and D_FSI_, (**b**) D_Li_ and D_TFSI_, and (**c**) D_Li_ and D_FSI_. Open circles represent the electrolytes of EMIFSI series.

The relationship between the diffusion coefficients appears in Figure 7. In Figure 7a, D_TFSI_ vs. D_FSI_ is represented. A straight line can be fitted irrespective of the type of ionic liquid, salt concentration, amount of sepiolite, etc., the slope of which is the ratio $\frac{r_{h_{FSI}}}{r_{h_{TFSI}}}$, if the Stokes–Einstein equation holds (Equation (3)): $D_{i}=\frac{\mathrm{kT}}{c\pi }\left(\frac{1}{r_{h_{i}}}\right)\left(\frac{1}{\eta }\right)$, where k is the Boltzmann constant (1.38 × 10^−23^ J·K^−1^), T is the absolute temperature, c is a constant with a proposed value of 5 [25], η is the liquid viscosity, and r is the effective Stokes radius. Then, $r_{h_{FSI}}=0.8\times r_{h_{TFSI}}$, a value frequently found in electrolytes containing a high concentration of these anions [26].

Figure 7b,c show the relationship between D_Li_ and D_TFSI_ or D_FSI_, respectively. Those prepared with EMIFSI fit a line with a similar slope but a slightly higher intercept than the rest of the electrolytes. As a consequence, D_Li_ is always slightly higher for the same D_TFSI_ in EMIFSI electrolytes. This seems not to have important practical consequences, because though systematic the difference is small. That the slope is very similar (*m* ≈ 0.1 for D_Li_ vs. D_TFSI_, and *m* = 0.07 for D_Li_ vs. D_FSI_) implies that $\frac{r_{h_{TFSI}}}{r_{h_{Li}}}$ and $\frac{r_{h_{FSI}}}{r_{h_{Li}}}$ are roughly the same, independent of the ionic liquid used; $\frac{r_{h_{TFSI}}}{r_{h_{Li}}}\approx 0.1$ and $\frac{r_{h_{FSI}}}{r_{h_{Li}}}=0.07$. This very high $r_{h_{Li}}$ as compared to the anions’ is caused by the complexation of Li by the oxyethylenic units and its transport along the PEO chain. That Li moves mostly through the PEO chain provides an explanation for the slightly higher values of D_Li_ in EMIFSI electrolytes, as, being PEO less crystalline and better mixed in them, Li’s transport along the polymer chain is less tortuous. 

**Figure 8:**
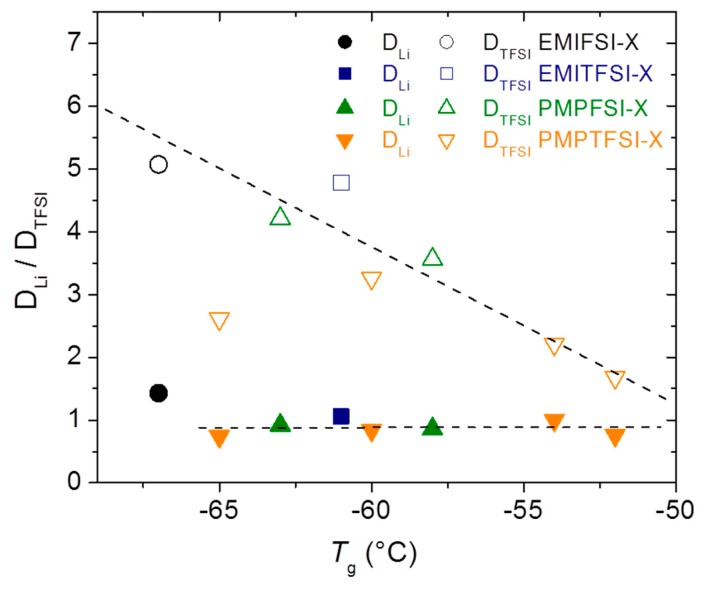
Variation of D_Li_ and D_TFSI_ as a function of the electrolytes’ *T*_g_.

Figure 8 shows the relationship of the electrolytes’ *T*_g_ with D_Li_ and D_TFSI_. Only electrolytes in which a defined *T*_g_ is observed in DSC traces (see Table 2) have been used to prepare this graph. It is noteworthy that for D_TFSI_ a single dependence with *T*_g_ is found irrespective of the RTIL nature, resembling what was shown in Figure 5b, where it was evidenced that σ depends more on the [RTIL] than on its nature. It is also remarkable that a shift of 15 °C in *T*_g_ makes D_TFSI_ vary by about 3-fold. Note that PMPTFSI-3, for which D_TFSI_ is not in the general trend, is much more crystalline than the rest of the electrolytes made with PMPTFSI, probably because of defective mixing. As for D_Li_, it is surprising that even though evidence that Li is moving through the PEO chain is seen along the work, its value seems to be independent of the *T*_g_ values. 

The complex blends presented in this work exemplify the extraordinary potential of polymer-based materials as electrolytes because of their unique combination of electrical properties, excellent mechanical properties, sustainability, and simple scalability. Especially remarkable is the diffusivity of the anions FSI and TFSI at 25 °C, some of them even over 10^−11^ m^2^·s^−1^, which evidences high potential for efficient anion transport..

## 4. Conclusions

Thermoplastic solid polymer electrolytes have been prepared in a laboratory extruder, in the absence of solvents, by melt compounding PEO, Li salts, and several RTILs together with TPGS-S acting as physical cross-linker of PEO. These blends can be considered safe electrolytes because of their solid state, which avoids leaks, and the low vapor pressure and non-flammability of the RTILs employed. They can also be considered sustainable electrolytes because of the absence of solvents in their processing, and because of two features characteristic of polymer-based materials: the low extrusion temperature and short processing times employed; and their thermoplasticity, which allows for recycling and reshaping. The effect of processing has been studied and shows that even very short residence times lead to effective solid-like electrolytes with small variations of σ. Increasing amounts of RTIL have been employed, leading to increasing σ. Values of about 2 × 10^−3^ S·cm^−1^ at 25 °C, and close to 1 × 10^−2^ S·cm^−1^ at 70 °C are attained, very remarkable indeed for solid electrolytes. High diffusivity of the anions FSI and TFSI at 25 °C, even over 10^−11^ m^2^·s^−1^, is seen, and evidences high potential for efficient anion transport. Though the excellent performance of TPGS-S as a physical cross-linker of PEO makes it plausible that an even higher concentration of ionic liquid could be employed while keeping the material a solid, the decay of ionic conductivity over a given concentration threshold suggests that the electrolytes presented in this work are close to the σ limit. The four RTILs employed, EMIFSI, EMITFSI, PMPFSI, and PMPTFSI, produce similar outcomes, and [RTIL] is the factor most affecting σ. However, EMIFSI makes the PEO crystallization more difficult than the other three RTILs and D_Li_ is slightly higher in electrolytes prepared with it. This work shows that the concept of a thermoplastic, extrudable electrolyte is possible and extremely appealing, and that the electrolytes’ formulations can be adapted to specific requirements in different energy storage devices, such as capacitors, supercapacitors, or batteries.

## Figures and Tables

**Table 1:** Composition and processing conditions (extrusion, temperature, residence time, and premixing stage) of the electrolytes.

Sample	[RTIL] (mol·m^−3^)	[LiTFSI] (mol·m^−3^)	[PEO] (mol·m^−3^)	TPGS-S (wt %)	*T* (°C)	Residence Time (min)	Premixing
**EMIFSI**							
EMIFSI-1	1323	566	16,504	5	140	20	NO
EMIFSI-2	1654	1011	12,008	2.5	120	20	NO
EMIFSI-3a	2285	755	9793	5	120	20	NO
EMIFSI-3b	2267	787	9714	5	120	20	NO
EMIFSI-3c	2267	787	9714	5	140	20	YES
EMIFSI-3d	2267	787	9714	5 *	160	20	YES
EMIFSI-3e	2253	809	9669	2.5	120	20	NO
EMIFSI-3f	2266	787	9719	2.5	160	20	YES
EMIFSI-3g	2267	782	9739	2.5	160	4	YES
EMIFSI-4	2857	0	9279	5	120	20	NO
**EMITFSI**							
EMITFSI-1	1724	0	15,297	2.5	120	20	NO
EMITFSI-2	1706	810	9853	2.5	120	20	NO
**PMPFSI**							
PMPFSI-1	1517	983	11,803	2.5	120	20	NO
PMPFSI-2	1524	1001	11,722	2.5	120	20	NO
PMPFSI-3	1603	1036	10,926	2.5	120	20	NO
PMPFSI-4	2062	0	14,423	2.5	120	20	NO
PMPFSI-5	2066	782	9465	2.5	120	20	NO
PMPFSI-6	2917	0	9001	5	120	20	NO
**PMPTFSI**							
PMPTFSI-1	894	1124	13,532	5	120	20	NO
PMPTFSI-2	1161	1002	12,030	2.5	120	20	NO
PMPTFSI-3	1249	612	13,575	5	120	20	NO
PMPTFSI-4	1600	802	9556	2.5	120	20	NO

* Pure sepiolite instead of TPGS-S.

**Table 2:** Physicochemical characterization of the electrolytes.

Sample	χ_c_ (%)	*T*_g_ (°C)	Rheology (75 °C)		Conductivity (25 °C) 10^3^ (S·cm^−1^)			α	Diffusivity (25 °C) (m^2^·s^−1^)		
			G′ = G″ (rad·s^−1^)	G′_0.05 rad·s^−1^_ (kPa)	σ	σ_NE_ (Li)	σ_NE_		10^12^ D_Li_	10^12^ D_TFSI_	10^12^ D_FSI_
**EMIFSI**											
EMIFSI-1	11	ns	0.02	8.49	0.56						
EMIFSI-2	14	−67			0.82	0.05	0.98	0.84	1.43	5.07	5.65
EMIFSI-3a	8	ns	ns	27.9	1.6	0.05	2.15	0.75	1.90	7.84	10.40
EMIFSI-3b	8	−64	0.03	7.29	1.7						
EMIFSI-3c	3	ns	ns	20.3	1.45	0.05		0.83	1.60	6.78	8.37
EMIFSI-3d	2	ns	0.02	5.63	1.74						
EMIFSI-3e	6	ns		29.8	1.6	0.06	2.35	0.69	1.85	8.55	11.46
EMIFSI-3f	9	ns	ns	23.2	1.73	0.06	2.54	0.68	1.90	8.70	12.46
EMIFSI-3g		?/−41 *		19.0	1.69						
EMIFSI-4	-	−70	ns	16.4	2.9						
**EMITFSI**											
EMITFSI-1	28	ns			0.5						
EMITFSI-2	-	−61			-	0.03	0.48		1.06	4.78	-
**PMPFSI**											
PMPFSI-1	24	−58			0.46	0.03	0.58	1.11	0.86	3.56	3.89
PMPFSI-2	21	−63			0.62						
PMPFSI-3	23	−63			0.66	0.04	0.88	0.75	0.92	4.21	5.95
PMPFSI-4	29	−57	0.02	5.84	0.72						
PMPFSI-5	11	ns	0.01	9.12	1.2	0.03	1.24	0.97	1.10	5.81	7.05
PMPFSI-6	18	ns	ns	13.4	1.2						
**PMPTFSI**											
PMPTFSI-1	7	−52			0.26	0.03	0.21	1.24	0.76	1.68	-
PMPTFSI-2	9	−54			0.3	0.04	0.31	0.96	0.99	2.21	-
PMPTFSI-3	22	−65			0.29	0.02	0.32	0.90	0.74	2.61	-
PMPTFSI-4	3	−60	0.02	2.18	0.8	0.03	0.49	1.61	0.84	3.26	-

ns: Not seen; * several seen.

**Table 3:** Viscosity at 25 °C (η_25 °C_) and melting temperature (*T*_m_) of the pure ionic liquids and of the solutions of LiTFSI in the ionic liquids employed (model liquid phases).

RTIL	LiTFSI wt %	PEG wt %	η_25 °C_ (mPa·s)	*T*_m_ (°C)
EMIFSI	-	-	24.5	−13
EMIFSI/LiTFSI	26	-	43.7	not seen
EMIFSI/LiTFSI/PEG	17.5	32.5	104	not seen
EMITFSI	-	-	35.5	−16
EMITFSI/LiTFSI	26	-	246	not seen
EMIFSI/LiTFSI/PEG	17.5	32.5	154	not seen
PMPFSI	-	-	52.7	−9.5
PMPFSI/LiTFSI	26	-	123	not seen
PMPFSI/LiTFSI/PEG	17.5	32.5	134	not seen
PMPTFSI	-	-	71.2	12
PMPTFSI/LiTFSI	26	-	790	−18 to 8
PMPTFSI/LiTFSI/PEG	17.5	32.5	158	not seen

**Table 4:** Vogel–Fulcher parameters calculated from the variation of the conductivity of the electrolytes with temperature.

Electrolyte	σ_∞_ (S·cm^−1^)	B (K)	*T*_0_ (K)	R^2^
EMIFSI-3g	1.70	885	171	1.000
EMIFSI-3f	1.75	904	169	0.999
EMIFSI-3c	1.76	919	168	0.999
EMIFSI-4	0.76	651	179	0.996
PMPFSI-5	1.07	881	168	0.999
